# Chronic disease associated with bullous pemphigoid risk: A systematic review and meta-analysis

**DOI:** 10.1016/j.jdin.2024.08.010

**Published:** 2024-09-07

**Authors:** Noppachai Siranart, Yanisa Chumpangern, Somkiat Phutinart, Patavee Pajareya, Rinrada Worapongpaiboon, Chanissara Winson, Charat Thongprayoon, Wisit Cheungpasitporn

**Affiliations:** a Department of Medicine, Mayo Clinic, Rochester, Minnesota; b Department of Medicine, Chulalongkorn University, Bangkok, Thailand; c Faculty of Medicine, Department of Medicine, Siriraj Hospital, Mahidol University, Bangkok, Thailand

**Keywords:** bullous pemphigoid, chronic disease, mortality, risk

## Abstract

**Background:**

Bullous pemphigoid (BP) is a chronic autoimmune blistering disease prevalent in the elderly, often accompanied by renal comorbidities. Immune dysregulation can lead to secondary BP and increased mortality rates in those already diagnosed.

**Methods:**

A literature review identified studies on the association between kidney disease and other comorbidities with BP. Pooled effect estimates were analyzed utilizing a random-effects model.

**Objective:**

To assess comorbidity risks with BP and determine mortality risk among BP patients with comorbidities.

**Results:**

Analysis included 45,323 BP patients from 49 studies. Kidney diseases were significantly linked to higher BP incidence (subdistribution hazard ratio 1.51, 95% CI: 1.10-2.07) and increased mortality (hazard ratio 1.62, 95% CI: 1.13-2.32). Cerebrovascular diseases, dementia, and diabetes also showed significant associations with both increased BP incidence and mortality (*P* < .05). However, cardiovascular diseases and malignancy were only associated with increased mortality among BP patients (*P* < .001) without affecting BP incidence (*P* = .785 and *P* = .792, respectively).

**Limitation:**

The study comprises mostly case-control, prospective, and retrospective observational studies, alongside data heterogeneity.

**Conclusion:**

This study reveals the association of several chronic conditions, including kidney diseases, with BP, contributing to elevated mortality rates. The findings emphasize the importance of management targeting both BP and associated comorbidities to improve patient outcomes.

**Capsule Summary**

• Bullous pemphigoid is a chronic blistering skin condition that often affects older adults.
• Kidney issues were found to be strongly linked to bullous pemphigoid with a higher chance of mortality. It is important for practitioners to treat both bullous pemphigoid and kidney comorbidities to enhance patient outcomes.

## Introduction

Bullous pemphigoid (BP), an autoimmune disease, is associated with morbidity. It typically manifests with pruritic papules and plaques, often accompanied by tense blisters. Studies across various populations estimate an incidence rate ranging from 2.4 to 42.8 per million per year, with the highest rates observed in European countries.^1^ The implications of BP go beyond its superficial manifestations, encompassing increased morbidity attributed to its persistency, the potential for intense pruritus, susceptibility to secondary skin infections, and a notable mortality risk, especially in the presence of comorbidities.^1^ The mechanism underlying the pathogenesis of BP is binding of autoantibodies that triggers an inflammatory cascade leading to complement activation, recruitment of inflammatory cells, and the release of proteolytic enzymes.^2^, ^3^, ^4^, ^5^ Previous case reports and case series have indicated that kidney diseases may contribute to secondary BP and elevate mortality risk in BP patients. This association is presumed to be linked to immune and complement activation, which can trigger flare-ups in individuals with BP.^6^^,^^7^

Moreover, previous studies showed various comorbidities affected the prognosis of BP patients, including cardiovascular diseases, cerebrovascular disease, and malignancy.^8^, ^9^, ^10^ These associations were also linked to BP, presuming an immunological process as the underlying mechanism. The primary objectives of this study are to (1) identify the risk of chronic diseases associated with BP, (2) investigate chronic diseases contributing to the development of BP, and (3) assess the risks of chronic disease leading to all-cause mortality among individuals with BP. The secondary objectives of this study aim to explore other comorbidities associated with BP and mortality.

## Methods

### Literature review and search strategy

Our systematic review and meta-analysis protocol has been registered with PROSPERO (International Prospective Register of Systematic Reviews; CRD NO. 42024520090). We conducted a systematic literature search, covering studies published up to January 2024 in databases including MEDLINE (via PubMed), EMBASE (via Scopus), and the Cochrane Database of Systematic Reviews. Our objective was to identify research focusing on the BP and comorbidities including kidney diseases, hypertension, dyslipidemia, coronary artery diseases, diabetes, chronic liver diseases, neurologic diseases, and malignancy.

To achieve this, 2 investigators, N.S. and W.C., independently compiled the systematic literature review using a designed search strategy. This strategy included terms such as "Bullous pemphigoid" alongside either "prognosis" or "mortality" or "morbidity" or "risk." There were no language restrictions. Additionally, we manually searched the reference lists of identified studies to further enrich our relevant articles. Throughout our systematic review and subsequent meta-analysis, we adhered to the Meta-analyses Of Observational Studies in Epidemiology standards and the Preferred Reporting Items for Systematic Reviews and Meta-Analyses statement.

### Selection criteria

Criteria for study selection included randomized controlled trials, cross-sectional studies, case-control studies, or cohort studies assessing the outcomes of the association between BP and comorbidities. Exclusion criteria comprised case reports and case series.

Selected studies were required to provide statistical outcomes in the form of mean ± SD or median (interquartile range Q1-Q3) with corresponding *P* values indicating significance in statistical hypothesis testing. Odds ratios (ORs) and hazard ratios (HRs), along with their 95% CIs, were analyzed to pool the estimated effects. Sample size and ethnicity of the population were not limiting factors for inclusion.

Quality assessment of included studies was conducted using the Newcastle-Ottawa quality assessment scale for case-control studies and the modified Newcastle-Ottawa scale for cohort and cross-sectional studies. Assessment encompassed 3 domains: study group selection (S) consisting of 4 items, group comparability (C) with 2 items, and exposure and outcome (O) including 3 items. Each domain received a maximum score of 4, 2, and 3, respectively, with bias assessment results presented as numerical values.

### Data abstraction

Using a structured data record form, the following information was collected from, including studies:

1. Basic information of literature: title, year of the study, name of the first author, publication year, and the country where the study was conducted
2. Patient baseline characteristics, demographic data, underlying diseases, and included population
3. OR and HR with 95% CI of the outcomes of interests

### Statistical analysis

The data analysis was conducted using R program (version 4.3.1). Adjusted point estimates were calculated from each study using DerSimonian and Laird's generic inverse variance technique, which assigned weights to studies based on their variance. To assess variation in prevalence across studies, Cochran's Q test was employed. In cases where heterogeneity was detected (*P* < .1 or I2 > 25%), the DerSimonian and Laird technique was used to apply an adjusted point estimate; otherwise, an inverse variance fixed-effect model was utilized. Publication bias was evaluated using the Egger’s test to determine whether there was evidence of bias in the included studies.

## Results

**Fig 1:**
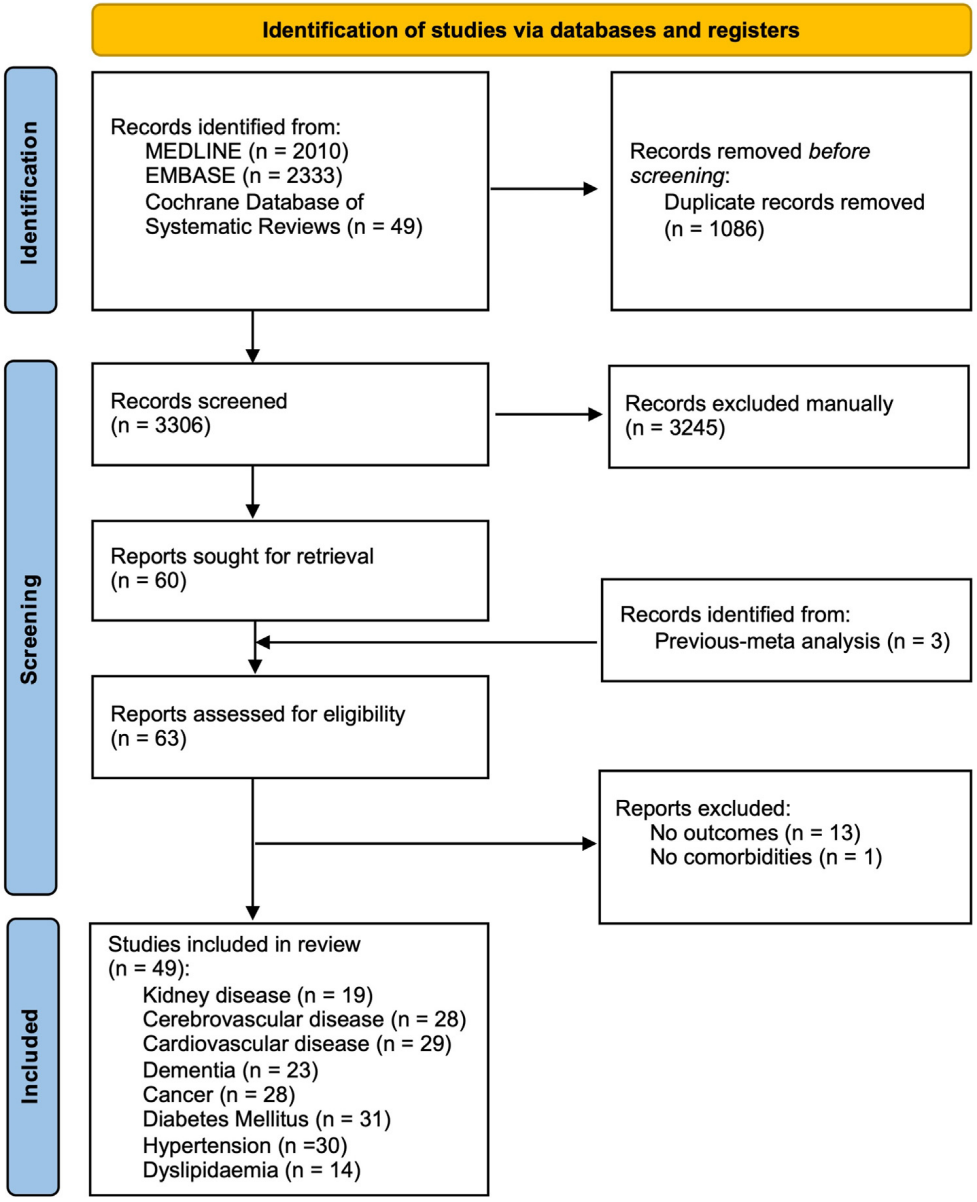
PRISMA flow diagram. The diagram delineates the thorough search approach divided into 3 sections: First, identifying studies through databases and registries; second, manually screening based on titles, abstracts, and eligibility criteria; and finally, categorizing included studies by comorbidities. *PRISMA*, Preferred Reporting Items for Systematic Reviews and Meta-Analyses.

After filtering out duplicate studies, our search strategy yielded 3306 articles. After screening the abstracts, 3245 studies that were not relevant were omitted. Hence, a total of 60 studies underwent further revision. Additionally, 3 studies were found through citation searches of previous meta-analyses, bringing the total number of studies reviewed to 63. The remaining 14 studies were later excluded due to failure to report outcomes or comorbidities of interest. As a result, the final analysis involved 49 studies evaluating 45,179 individuals diagnosed with BP (Table I). These chosen studies were divided into 3 categories: kidney diseases and other associated conditions related to BP, kidney diseases and other associated conditions linked to the incidence of BP development, and kidney diseases and other associated conditions linked to mortality in individuals with BP. Fig 1 shows the literature review's inclusion and exclusion process.

**Table I:** Baseline characteristics of all included studies

Outcome	Author	Year	Single/multicenter	Study period	Study design	Study population	*N* (BP)	Match	Age	Female, *n* (%)	Follow-up years	Groups	Quality
1	Bastuji-Garin et al^20^	2010	Multicenter	Jan 2003 to Apr 2007	Prospective case-control	BP diagnose with clinical features, pathology with DIF	201	Age, sex, center, place of residence	82.4 ± 8.7	130 (64.7)	N/A	Cerebrovascular disease, DM	S4C2E3
1	Brick et al^21^	2014	Multicenter	Jan 1960 to Dec 2009	Retrospective case-control	BP diagnose with clinical features, IIF, pathology with DIF	87	Age, sex	77.5	50 (57)	N/A	Cerebrovascular disease, dementia	S4C2E3
1	Casas-de-la-Asunción et al^22^	2014	Single	Jan 2002 to Feb 2012	Retrospective case-control	BP severity was classified as generalized (affecting 2 or more anatomic locations) or localized.	54	Age, sex	80.8	34 (60.7)	N/A	CA, cerebrovascular disease, CVD, dementia, DM	S4C2E3
1	Chen et al^23^	2011	Single	Jan 1997 to Dec 2008	Retrospective case-control	BP diagnose with codes in database	3485	Age, sex	74	1909 (54.8)	N/A	Cerebrovascular disease, dementia	S4C2E3
1	Daneshpazhooh et al^24^	2017	Single	Jan 2006 to Dec 2011	Retrospective case-control	BP diagnose with clinical features, IIF, pathology with DIF	160	Age, sex	68.8 ± 18.6	75 (46)	N/A	Cerebrovascular disease	S4C2E3
1	Jedlickova et al^25^	2010	Single	Jan 1991 to Dec 2006	Retrospective case-control	BP diagnose with clinical features, IIF, pathology with DIF	89	Age, sex	76 ± 8.7	45 (50.6)	N/A	CA, CVD, DM	S4C2E3
1	Kalińska-Bienias et al^26^	2019	Single	Jan 2000 to Dec 2014	Retrospective case-control	BP diagnose with clinical features, IIF	218	Age, sex	76.2 ± 11.6	137 (62.8)	N/A	CA, cerebrovascular disease, CVD, dementia, DM, HTN	S4C2E3
1	Kibsgaard et al^27^	2017	Single	Jan 1977 to Jan 2015	Retrospective case-control	BP diagnose with codes in database	3281	Age, sex	76.5 ± 12.6	1447 (44.1)	N/A	CA, cerebrovascular disease, CVD, DM, HTN	S4C2E3
1	Kwa et al^29^	2017	Single	Jan 2002 to Dec 2012	Retrospective case-control	BP	13,342	Age, sex, race/ethnicity, mean annual household income, insurance status, number of chronic conditions, hospital region	N/A	N/A	N/A	Cerebrovascular disease, CVD, DM, HTN	S4C2E3
1	Kwan et al^12^	2015	Single	Jan 2004 to Dec 2013	Retrospective case-control	BP diagnose with clinical features, IIF, pathology with DIF	43	Age, sex, ethinicity	79.4	19 (44.2)	N/A	CA, cerebrovascular disease, CVD, DLP, DM, HTN, KD	S4C2E3
1	Langan et al^13^	2010	Multicenter	Jan 1996 to Dec 2006	Retrospective case-control	BP diagnose with codes in database	868	Age, sex, general practice	80	534 (62)	N/A	CA, cerebrovascular disease, dementia, KD	S4C2E3
1	Lee et al^14^	2020	Single	Jan 2001 to Nov 2014	Retrospective case-control	BP diagnose with clinical features	91	Age, sex	76	40 (53)	N/A	CVD, DLP, DM, HTN, KD	S4C2E3
1	Martin et al^15^	2021	Single	June 2002 and May 2013	Retrospective case-control	BP diagnose with clinical features, pathology with DIF	300	Age, sex	77.9 ± 11.0	136 (45.3)	N/A	Cerebrovascular disease, DM, KD	S4C2E3
1	Pankakoski et al^30^	2018	Single	Jan 2012 to Dec 2013	Retrospective case-control	BP diagnose with clinical features, IIF, pathology with DIF	70	Age	77.1	34 (48.6)	N/A	Cerebrovascular disease, CVD, DM, HTN	S4C1E3
1	Papakonstantinou et al^31^	2019	Single	Jan 2011 to Dec 2015	Retrospective case-control	BP diagnose with codes in database	183	Age, sex	80 ± 12	125 (68)	N/A	Cerebrovascular disease, dementia	S4C2E3
1	Phuan et al^32^	2017	Single	Jan 2010 to May 2015	Retrospective case-control	BP diagnose with clinical features, IIF	103	Age, sex	78.1	50 (48.5)	N/A	Cerebrovascular disease, dementia	S4C2E3
1	Ren et al^16^	2017	Single	Jan 2002 to Dec 2012	Cross-sectional	-	N/A	N/A	N/A	(37.7)	N/A	CVD, DM, HTN, KD	S4C2E3
1	Ren et al^17^	2017	Single	Jan 2002 to Dec 2012	Cross-sectional	BP diagnose with codes in database	2105	Age, sex, race	76.7 ± 0.2	1280 (60.8)	N/A	CA, CVD, dementia, DM, HTN, KD, LD	S4C2E3
1	Sayar et al^28^	2021	Single	Jan 1987 to Dec 2017	Retrospective case-control	BP diagnose with clinical features, IIF	145	Age, sex	66.4 ± 15.4	79 (54.5)	N/A	CA, cerebrovascular disease, CVD, dementia, DM, HTN	S4C2E3
1	Sim et al^18^	2017	Single	Jan 2005 to Dec 2014	Retrospective case-control	BP diagnose with clinical features	105	Age, sex	78 ± 11	54 (51)	N/A	CA, DLP, DM, HTN, KD	S4C2E3
1	Stone and Schroeter^33^	1975	Single	Jan 1960 to Dec 1972	Retrospective case-control	BP diagnose with clinical features	73	Age, sex, calendar year of diagnosis	66	38 (52.1)	N/A	CA	S4C2E3
1	Teixeira et al^34^	2014	Single	Jan 1998 to Dec 2010	Retrospective case-control	BP diagnose with clinical features	77	Age, sex	79.6	39 (50.6)	N/A	Cerebrovascular disease, dementia, HTN	S4C2E3
1	Venning et al^35^	1990	Single	Jan 1975 to Dec 1989	Retrospective case-control	BP diagnose with clinical features, IIF, pathology with DIF	84	Age, sex	73.9	45 (53.6)	N/A	CA	S4C2E3
1	Wu et al^19^	2023	Single	Jan 2000 to Dec 2013	Retrospective case-control	BP diagnose with codes in database	9344	Age, sex	75.9 ± 13.9	5050 (54)	N/A	Cerebrovascular disease, CVD, dementia, DLP, DM, HTN, KD, LD	S4C2E3
1	Zhang et al^36^	2022	Single	Jan 2009 to May 2021	Retrospective case-control	BP diagnose with clinical features, IIF, pathology with DIF	162	Age, sex	68	75 (46.3)	N/A	DLP, HTN	S4C2E3
2	Kridin et al^43^	2021	Single	Jan 2002 to Dec 2019	Retrospective cohort	(i) Documented diagnosis of BP registered at least twice by a board-certified dermatologist; or (ii) Diagnosis of BP in discharge letters of patients admitted to dermatological wards.	3294	Age, sex, race	76.7	1627 (57.5)	3.4	CA	S4C2O2
2	Ma et al^38^	2022	Single	2007 to 2018	Retrospective cohort	Diabetic patients	108	Age, sex, duration	59.3 ± 11.8	198927 (44.5)	1.3 ± 0.6	CA, cerebrovascular disease, CVD, dementia, DLP, HTN, KD, LD	S4C2O2
2	Tang et al^39^	2023	Single	Jan 2008 to Dec 2019	Retrospective cohort	Patients with ESRD who underwent chronic hemodialysis or peritoneal dialysis	426	Age, sex, index	64.5 ± 14.1	43272 (46.3)	4.6	CA, CVD, DLP, DM, HTN, KD	S4C2O2
2	Chen et al^37^	2019	Single	Jan 2002 to Dec 2011	Retrospective cohort	Patients with cancer	193	Age, sex, index, date	61.2 ± 15.2	16746 (45.5)	10	CA, cerebrovascular disease, CVD, dementia, DLP, DM, HTN, KD, LD	S4C2O2
2	Wu et al^40^	2020	Single	Jan 2000 to Dec 2012	Retrospective cohort	Neurological cancer patients	24	Age, sex, index, date	49.2 ± 20.2	3562 (42.8)	12	CA, cerebrovascular disease, CVD, dementia, DLP, DM, HTN, KD, LD	S4C2O2
2	Wu et al^41^	2021	Single	Jan 2011 to Dec 2015	Retrospective cohort	Diabetic patients	91	Age, sex, duration of DM, insulin usage, propensity scores of comorbidities	52.4 ± 10.9	44434 (35.7)	4.3 ± 3.0	CVD, DLP, HTN, KD, LD	S4C2O2
2	Yu et al^42^	2023	multicenter	Jan 2007 to Dec 2018	Retrospective cohort	CKD and non-CKD patients	1737	Age, sex, comorbidities	69.2 ± 13.4	269171 (42.2)	3.0 ± 1.8	CA, cerebrovascular disease, CVD, dementia, DLP, DM, HTN, KD, LD	S4C2O2
3	Amonchaisakda et al^48^	2020	Single	Jan 2007 to Dec 2016	Retrospective cohort	BP diagnose with pathology with DIF	119	N/A	82	59 (49.6)	1.67	DM, HTN	S4C2O2
3	Cai et al^44^	2013	Single	Apr 2004 to Dec 2009	Retrospective cohort	BP diagnose with clinical features, IIF, pathology with DIF	359	Age, sex	75.7 ± 2.6	187 (52.1)	2.1	CA, cerebrovascular disease, CVD, dementia, DM, HTN, KD, LD	S4C2O2
3	Cortés et al^49^	2011	Single	Jan 2001 to Dec 2022	Prospective cohort	BP diagnose with clinical features, IIF, pathology with DIF	115	Age, sex	76.4 ± 15.3	68 (59.1)	3	CA, CVD, DM, LD	S4C2O2
3	Cortés et al^50^	2012	Single	Jan 1990 to Dec 2003	Retrospective cohort	BP diagnose with clinical features, IIF, pathology with DIF	60	N/A	79.5 ± 11.6	34 (56.7)	2.8 ± 2.3	CVD, dementia, DM, HTN	S4C2O2
3	Försti A-K. et al^51^	2016	Multicenter	Jan 1985 to Dec 2012	Retrospective cohort	BP diagnose with pathology with DIF	198	Age, sex	77.5 ± 10.4	102 (51.5)	N/A	CA	S4C2O2
3	Gual et al^45^	2014	Single	Jan 1990 to Dec 2010	Retrospective cohort	BP patient	101	N/A	77.8	49 (48.5)	1	CA, CVD, DLP, DM, HTN, KD	S4C2O2
3	Lee et al^46^	2014	Single	Jan 1993 to Dec 2013	Retrospective cohort	BP diagnose with clinical features, IIF, pathology with DIF	168	Age	69.2 ± 15.7	84 (50)	2.5	CA, cerebrovascular disease, CVD, dementia, DM, HTN	S4C2O2
3	Li et al^52^	2014	Single	Jan 1991 to Jan 2011	Retrospective cohort	BP diagnose with pathology with DIF	140	Age	64.3 ± 13.6	58 (41.4)	3	CA, CVD, DM, HTN, KD	S4C2O2
3	Monshi et al^53^	2020	Single	Oct 2001 to Jan 2012	Retrospective cohort	BP diagnose with IIF	100	Age, sex	81	56 (56)	5	CA, cerebrovascular disease, CVD, dementia, DM, HTN	S4C2O2
3	Papara et al^54^	2023	Single	July 2001 to Nov 2019	Retrospective cohort	BP diagnose with clinical features, pathology with DIF	148	N/A	74.5	80 (54)	2	CA, cerebrovascular disease, CVD, dementia	S4C2O2
3	Roujeau et al^55^	1998	Multicenter	Jan 1985 to Dec 1991	Retrospective cohort	BP diagnose with clinical features, IIF, pathology with DIF	217	N/A	79 ± 11	120	0.5	Dementia	S4C2O2
3	Rozenblat et al^56^	2019	Single	Jan 2009 to Dec 2016	Retrospective cohort	BP diagnose with clinical features, IIF, pathology with DIF	87	Age, sex	79.1	40 (46)	1	Dementia	S4C2O2
3	Shen et al^57^	2022	Single	Mar 2019 to Apr 2021	Retrospective cohort	BP diagnose with clinical features, pathology with DIF	252	Age, sex, date of the dermatology clinic visit	78	115 (45.6)	4	CA, CVD	S4C2O1
3	Titou et al^58^	2022	Single	Jan 2008 to Dec 2017	Retrospective cohort	BP diagnose with clinical features, IIF, pathology with DIF	93	Age, sex	71.1 ± 11.3	42 (45.2)	27 ± 14.1	CA, DLP, DM, HTN	S4C2O2
3	Wu et al^41^	2021	Multi center	Jan 1997 to Dec 2013	Retrospective cohort	BP diagnose with codes in database	2260	Age, sex, propensity score of comorbidities, use of tetracycline	75.9 ± 10.4	970 (42.9)	3.1 ± 3.3	Cerebrovascular disease, CVD, DLP, DM, HTN, KD, LD	S4C2O2
3	Zhang et al^59^	2013	Single	Jan 2005 to May 2010	Prospective cohort	BP diagnose with histopathology, DIF and IIF	116	N/A	71 ± 12.6	41 (43.6)	2.7	Cerebrovascular disease, CVD, DM, HTN	S4C2O3
1, 3	Jeon et al^11^	2018	Single	Jan 2006 to Dec 2013	Retrospective cohort	BP diagnose with clinical features, IIF, pathology with DIF	103	Age, sex	74.4 ± 10.6	50 (48.5)	47.1 ± 32.2	CA, cerebrovascular disease, dementia, DM, HTN, KD, LD	S4C2O2

*BP*, Bullous pemphigoid; *CA*, cancer; *CKD*, chronic kidney disease; *CVD*, cardiovascular disease; *DIF*, direct immunofluorescence; *DLP*, dyslipidemia; *DM*, diabetes mellitus; *HTN*, hypertension; *IIF*, indirect immunofluorescence; *KD*, kidney disease; *LD*, liver disease.

### Outcome 1: Kidney diseases and other comorbidities associated with BP

#### Kidney diseases

A total of 9 studies^11^, ^12^, ^13^, ^14^, ^15^, ^16^, ^17^, ^18^, ^19^ involving 12,959 BP patients were included to analyze the association between kidney diseases and BP. No significant association was observed in both univariable (HR 1.12, 95% CI: 0.51-2.46, *P* = .712) and multivariable analyses (HR 2.10, 95% CI: 0.43-10.29, *P* = .284). However, substantial heterogeneity was observed for both analyses (I2 = 84%, *P* < .01 and I2 = 91%, *P* < .01 for univariable and multivariable analysis, respectively) (Table II).

**Table II:** Bullous pemphigoid comorbidities

Comorbidity	Univariate		Multivariate	
	OR (95% CI)	*P* value	OR (95% CI)	*P* value
CA	1.20 (0.75-1.92)	.391	1.45 (0.27-7.75)	.444
Cerebrovascular disease	2.43 (1.88-3.14)	<.001	2.38 (1.56-3.63)	.001
Cardiovascular disease	1.41 (1.21-1.65)	.002	1.04 (0.68-1.60)	.806
Dementia	4.71 (3.40-6.53)	<.001	3.43 (2.43-4.86)	<.001
DLP	0.56 (0.25-1.28)	.094	0.88 (0.24-3.21)	.716
DM	1.60 (1.17-2.19)	.007	1.57 (0.95-2.57)	.069
HTN	1.21 (0.77-1.91)	.361	1.76 (0.91-3.40)	.083
KD	1.12 (0.51-2.46)	.712	2.1 (0.43-10.29)	.284

Pooled estimate of odds ratio of association between comorbidities and bullous pemphigoid.

*CA*, Cancer; *DLP*, dyslipidemia; *DM*, diabetes mellitus; *HTN*, hypertension; *KD*, kidney disease; *OR*, odds ratio.

#### Other comorbidities

A total of 26 studies^11^, ^12^, ^13^, ^14^, ^15^, ^16^, ^17^, ^18^, ^19^, ^20^, ^21^, ^22^, ^23^, ^24^, ^25^, ^26^, ^27^, ^28^, ^29^, ^30^, ^31^, ^32^, ^33^, ^34^, ^35^, ^36^ involving 34,773 BP patients were included for analysis of the association between other comorbidities and BP. Cerebrovascular diseases and dementia demonstrated significant associations with increased odds of BP in both univariable (OR 2.43, 95% CI: 1.88-3.14 and OR 4.71, 95% CI: 3.40-6.53, respectively) and multivariable analyses (OR 2.38, 95% CI: 1.56-3.63 and OR 3.43, 95% CI: 2.43-4.86, respectively). In addition, increased odds of BP were also associated with cardiovascular diseases and diabetes in univariable analysis (OR 1.41, 95% CI: 1.21-1.65 and OR 1.60, 95% CI: 1.17-2.19, respectively) but not multivariable analysis (*P* = .806 and *P* = .069, respectively). Other conditions, including malignancy and dyslipidemia did not exhibit any association with odds of BP in both univariable (*P* = .391 and *P* = .094, respectively) and multivariable analyses (*P* = .444 and *P* = .716, respectively).

### Outcome 2: Kidney diseases and other comorbidities associated with development of BP

#### Kidney diseases

A total of 6 studies^37^, ^38^, ^39^, ^40^, ^41^, ^42^ involving 2579 BP patients were included for the analysis of the association between kidney diseases and BP development (Table III). Through multivariable analysis, kidney diseases were linked to an increased incidence of BP (sub-distribution hazard ratio [SDHR] 1.51, 95% CI: 1.10-2.07), with no significant heterogeneity detected among the pooled SDHRs (*P* = .10). However, due to the limited number of studies available, pooling of cause-specific HRs and HRs from univariable analysis was not feasible.

**Table III:** Bullous pemphigoid development

Comorbidity	Multivariate			
	Subdistrbution HR (95% CI)	*P* value	Cause-specific HR (95% CI)	*P* value
CA	0.91 (0.33-2.51)	.792	1.8 (0.06-55.95)	.540
Cerebrovascular disease	2.50 (2.20-2.84)	<.001		
Cardiovascular disease	1.01 (0.92-1.11)	.785		
Dementia	2.83 (1.06-7.51)	.043		
DLP	0.91 (0.72-1.15)	.319		
DM	1.61 (1.50-1.73)	.001		
HTN	1.15 (0.94-1.40)	.132		
KD	1.51 (1.10-2.07)	.023		
LD	0.95 (0.63-1.42)	.729		

Pooled estimate of hazards ratio of association between comorbidities and bullous pemphigoid development.

*CA*, Cancer; *DLP*, dyslipidemia; *DM*, diabetes mellitus; *HR*, hazard ratio; *HTN*, hypertension; *KD*, kidney disease; *LD*, liver disease.

#### Other comorbidities

A total of 7 studies^37^, ^38^, ^39^, ^40^, ^41^, ^42^, ^43^ involving 5873 BP patients were included for the analysis of the association between other comorbidities and BP development. Cerebrovascular diseases (SDHR 2.5, 95% CI: 2.20-2.84), dementia (SDHR 2.83, 95% CI: 1.06-7.51), and diabetes (SDHR 1.61, 95% CI: 1.50-1.73) displayed significant associations with increased incidence of BP in multivariable analysis. In contrast, malignancy (*P* = .792), cardiovascular diseases (*P* = .785), dyslipidemia (*P* = .319), hypertension (*P* = .132), and liver diseases (*P* = .729) were not found to be associated with BP development in multivariable analysis. HRs for univariable analysis and cause-specific HR were not pooled due to the limited number of studies included.

### Outcome 3: Kidney diseases and other comorbidities associated with mortality in patients with BP

#### Kidney diseases

A total of 5 studies^11^^,^^44^, ^45^, ^46^, ^47^ involving 2963 BP patients were included for the analysis of the association between kidney diseases and mortality in BP patients (Table IV). Kidney diseases were associated with increased mortality in univariable analysis (HR 1.62, 95% CI: 1.13-2.32) with no heterogeneity among the pooled HRs (*P* = .14). However, HRs from multivariable analysis of kidney diseases were not pooled due to the limited number of studies available.

**Table IV:** Mortality in bullous pemphigoid

Comorbidity	Univariate		Multivariate	
	HR (95% CI)	*P* value	HR (95% CI)	*P* value
CA	1.78 (1.37-2.32)	<.001	1.78 (1.17-2.71)	.019
Cerebrovascular disease	2.01 (1.79-2.25)	<.001	2.40 (1.05-5.45)	.043
Cardiovascular disease	2.37 (1.38-4.08)	.006	2.07 (1.34-3.20)	.005
Dementia	2.06 (1.48-2.88)	.003	1.99 (0.60-6.58)	.166
DLP	0.90 (0.77-1.06)	.113		
DM	1.26 (0.76-2.09)	.324	2.36 (1.02-5.45)	.047
HTN	1.19 (0.86-1.65)	.240	0.88 (0.42-1.87)	.638
KD	1.62 (1.13-2.32)	.020		

Pooled estimate of hazards ratio of association between comorbidities and mortality in bullous pemphigoid population.

*CA*, Cancer; *DLP*, dyslipidemia; *DM*, diabetes mellitus; *HR*, hazard ratio; *HTN*, hypertension; *KD*, kidney disease.

#### Other comorbidities

A total of 17 studies^11^^,^^44^, ^45^, ^46^, ^47^, ^48^, ^49^, ^50^, ^51^, ^52^, ^53^, ^54^, ^55^, ^56^, ^57^, ^58^, ^59^ involving 4636 BP patients were included for the analysis of the association between other comorbidities and mortality in BP patients. Malignancy, cerebrovascular diseases, and cardiovascular diseases also exhibited significant association with increased death in both univariable (HR 1.78, 95% CI: 1.37-2.32; HR 2.01, 95% CI: 1.79-2.25; HR 2.37, 95% CI: 1.38-4.08, respectively) and multivariable analyses (HR 1.78, 95% CI: 1.17-2.71; HR 2.4, 95% CI: 1.05-5.45; HR 2.07, 95% CI: 1.34-3.2, respectively). Additionally, dementia was associated with incidence of death in univariable analysis (HR 2.06, 95% CI: 1.48-2.88) but not multivariable analysis (*P* = .166), while diabetes showed significant association in multivariable analysis (HR 2.36, 95% CI: 1.02-5.45) but not univariable analysis (*P* = .324). Other conditions, including dyslipidemia (*P* = .113 for univariable analysis) and hypertension (*P* = .240 and .638 for univariable and multivariable analysis, respectively), did not exhibit significant association with mortality in BP. Pooling of HRs for multivariable analyses of dyslipidemia and kidney diseases was not conducted due to the limited number of studies.

## Discussion

This meta-analysis represents the first to demonstrate the risks associated with kidney diseases in the development of BP, as well as the subsequent increase in mortality among BP patients. Moreover, our study also revealed significant associations between BP and various comorbidities, including cerebrovascular diseases, dementia, and diabetes, highlighting their role in influencing all-cause mortality among BP patients.

Recent case reports and a case-control study have highlighted a relation between renal diseases and BP. Literature suggests a positive correlation in the incidence of immune disorders affecting the basement membranes of the skin and kidneys, with the severity of BP skin lesions often mirroring that of kidney disease. Additionally, there have been instances where BP has emerged secondary to kidney conditions such as membranous nephropathy. Chronic kidney disease may also predispose patients to renal infections, which could trigger a secondary BP flare due to immune system activation.^6^^,^^7^ Emerging evidence suggests that dysfunction of CD4+CD25+Foxp3+ regulatory T cells (Tregs) due to uremia-related factors in chronic kidney disease patients may disrupt peripheral immune tolerance, resulting in a progressive loss of immunosuppressive function, ultimately leading to BP.^60^, ^61^, ^62^

Diabetes is closely associated with chronic inflammation caused by disruption of immune function and heightens the risk of autoimmune disorders such as BP. Consequently, diabetic patients frequently experience more severe BP symptoms and may exhibit heightened resistance to treatment, potentially leading to increased mortality rates. Additionally, diabetes-induced alterations in skin properties, including decreased elasticity and increased fragility, could render the skin more susceptible to blistering, further exacerbating the association between diabetes and BP. Considering these interconnected factors, the observed association between diabetes and BP, along with its implications for increased mortality, becomes more comprehensible.^63^^,^^64^ Although corticosteroids remain the cornerstone of BP treatment, their potential to induce hyperglycemia is a significant consideration, especially in patients with concurrent health issues requiring prolonged treatment durations.^18^^,^^65^ Our research suggested the association between diabetes and heightened mortality risk among BP patients. Therefore, it is essential to carefully consider when administering systemic glucocorticoids to BP patients with diabetes, as it may lead to hyperglycemia and potentially elevate the risk of mortality.

The mechanism linking neurological diseases including cerebrovascular diseases and dementia with BP remains incompletely understood; nevertheless, a potential explanation involves autoimmunity targeting BP antigens present in both the brain and skin. This hypothesis suggests that autoimmunity against brain BP antigens, which cross-react with their skin counterparts, could serve as a triggering factor. Our findings are in concordance with prior studies, suggesting that a neuronal isoform of BP antigens may incite an autoimmune response against the epithelial isoform, contributing to BP development.^20^^,^^23^^,^^66^ Our study supported that neurological diseases can independently raise mortality rates in patients with BP. Hence, it is crucial to be cautious when managing individuals with BP alongside dementia or stroke.

This meta-analysis has several limitations. Firstly, the majority of the studies included are case-control, prospective, and retrospective observational studies, meaning that the value of the meta-analysis is limited. Therefore, it is important to recognize that a causal association cannot be definitively concluded. Secondly, high heterogeneity, despite efforts to address it, remains a major limitation of our study. This heterogeneity can stem from differences in study designs, patient populations, methodologies, and other factors, which may affect the consistency and generalizability of the results. However, despite these limitations, this study contributes valuable insights and expands our understanding of the association between BP and kidney diseases, as well as other comorbidities. Further well-designed studies are warranted to gain deeper insights into the mechanisms underlying our findings.

## Conclusion

Several chronic conditions including kidney diseases have been found to be associated with BP, which in turn contributes to elevated mortality rates among affected patients. These findings underscore the critical importance of comprehensive management strategies that not only aim to reduce the incidence of BP but also significantly mitigate the mortality risks associated with the disease and its comorbidities.

## Conflicts of interest

None disclosed.
